# Multimodal and Multistimuli 4D-Printed Magnetic Composite Liquid Crystal Elastomer Actuators

**DOI:** 10.1021/acsami.3c14607

**Published:** 2023-12-27

**Authors:** Erick R. Espíndola-Pérez, Javier Campo, Carlos Sánchez-Somolinos

**Affiliations:** † Departamento de Física de la Materia Condensada, Instituto de Nanociencia y Materiales de Aragón (INMA), CSIC-Universidad de Zaragoza, Zaragoza 50009, Spain; ‡ Centro de Investigación Biomédica en Red de Bioingeniería, Biomateriales y Nanomedicina, Instituto de Salud Carlos III, Zaragoza 50018, Spain

**Keywords:** liquid crystalline elastomers, 4D printing, magnetic soft robots, multimodal devices, multistimuli actuators

## Abstract

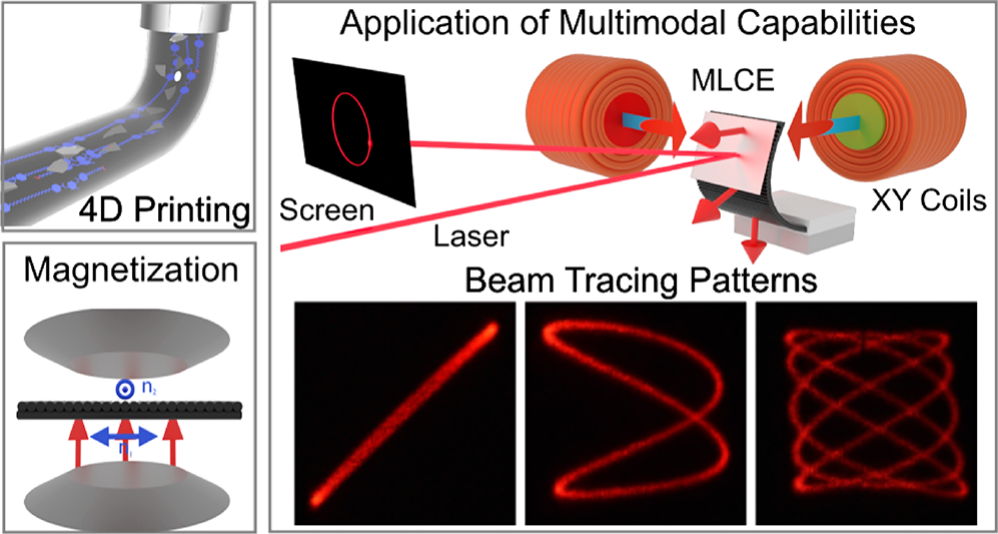

Liquid crystal elastomer (LCE)-based soft actuators are being studied for their significant shape-changing abilities when they are exposed to heat or light. Nevertheless, their relatively slow response compared with soft magnetic materials limits their application possibilities. Integration of magnetic responsiveness with LCEs has been previously attempted; however, the LCE response is typically jeopardized in high volumes of magnetic microparticles (MMPs). Here, a multistimuli, magnetically active LCE (MLCE), capable of producing programmable and multimodal actuation, is presented. The MLCE, composed of MMPs within an LCE matrix, is generated through extrusion-based 4D printing that enables digital control of mesogen orientation even at a 1:1 (LCE:MMPs) weight ratio, a challenging task to accomplish with other methods. The printed actuators can significantly deform when thermally actuated as well as exhibit fast response to magnetic fields. When combining thermal and magnetic stimuli, modes of actuation inaccessible with only one input are achieved. For instance, the actuator is reconfigured into various states by using the heat-mediated LCE response, followed by subsequent magnetic addressing. The multistimuli capabilities of the MLCE composite expand its applicability where common LCE actuators face limitations in speed and precision. To illustrate, a beam-steering device developed by using these materials is presented.

## Introduction

Magnetic elastomers (MEs) have drawn a great deal of attention in the field of soft robotics due to their advantageous properties including precise and untethered control, fast response, as well as their shape-changing capabilities.^1−8^ Common MEs are composed of magnetic microparticles (MMPs) based in iron, its oxides or alloys, which are embedded into elastomers like natural rubber, polyurethane, or silicone-based.^7,9−12^ Generally MEs derive their actuation properties solely from the magnetic nature of their solids, restraining their functionality to the magnetic moment of the particles or their distribution within the polymer matrix.^1,5,13,14^ Achieving complex patterns in MEs, needed for soft robotic functions, can be challenging or may require multiple processes.^15−19^

Another prominent example of responsive materials for the creation of soft robots are liquid crystal elastomers (LCEs).^20−23^ They consist of weakly cross-linked liquid crystalline (LC) polymeric systems. Their mechanical response comes from the loss of order of the LC molecules, known as mesogens, due to the energy input of stimuli.^21,24−26^ This process causes the polymer network to contract along the direction of the preferential orientation of the long molecular axes of the mesogens, known as the director *n*, and to expand along the perpendicular directions.^27−31^ Given this anisotropic mechanical response, it is crucial in these materials to have precise control of the director.^29,32−36^ Recently, four-dimensional (4D) printing emerged as a powerful tool to accurately define the director in LCE constructs.^23,37−40^ Mesogen alignment induced during ink extrusion and deposition by rheological means, combined with the digital control over the path of the extruding nozzle during three-dimensional (3D) printing, allows a precise definition of the final LCE morphology via computer-aided design (CAD).^23,37,40^ The resulting LCE objects are dynamic over time when a stimulus is applied. These actuators are capable of producing large strains upon the application of different stimuli, like heat,^23,33,37^ humidity,^41^ or light.^34,40,42^

Despite their ability to undergo significant and reversible anisotropic mechanical deformations,^21,22,27,29^ which sets them apart from other matrix materials, LCEs have not received extensive attention as matrix materials for MEs or magnetically actuated soft robots.^7,9,12,43−47^ Only a few examples in the literature^48−51^ make use of magnetic LCEs composites to provide shape-changing capabilities. However, such properties mostly rely on the magnetic actuation of the material, leaving the shape-morphing functionality offered by the composite’s LC characteristics, underexploited. Recently, films of magnetic liquid crystal elastomers with surface-programmed complex azimuthal director orientation have been successfully produced, along with a high concentration of MMPs.^50^ However, the shape-morphing response of the system when exposed to high temperature differs substantially from the natural response of the pure LCE without MMPs. The presence of these MMPs locally perturbs the director field and hinders the preparation of magnetically responsive LCEs (MLCEs) with well-defined director patterns by using alignment surfaces. Furthermore, the MLCE elements that are built within liquid crystal cells have a reduced ability to implement complex bulk director patterns or thicker elements beyond a few tens of microns. In addition, to produce elements of complex shapes, postprocessing such as mechanical deformations or cutting, for the elements to be adjusted to the desired geometry, need to be performed. Overall, there is a need to find suitable materials and processing methodologies to narrow the gap between these materials and real-world practical applications, in a way that could be scalable or automated.

In this work, we present a new composite mixture prepared from LC macromers and MMPs that is deposited by using a 3D printing platform, leading to elements with well-defined geometries and digitally controlled local anisotropy, even at high concentrations of MMPs. The complex morphology achieved is later fixed as an LCE by photopolymerization. This enables precise programming of the mechanical response when stimuli like temperature are applied. In addition to this mechanical deformation controlled by the LC properties, MMPs within the elastomer can also be subsequently magnetized through the application of a strong magnetic field (ranging from hundreds of mT to T), thereby providing them with magnetic responsiveness. After this process, the actuator can be wirelessly activated using a magnetic field with a significantly smaller magnitude (a few mT), offering a fast response mode.

The well-defined director morphology, established using 4D printing, enables one to leverage the temperature-responsive morphological transformation inherent in LCEs to showcase a multimodal actuator that responds to magnetic fields. By applying heat, the actuator can be reconfigured into a plurality of states that can then be addressed by using magnetic fields, offering access to a variety of well-defined deformations and actuation modes that are hardly accessible with conventional MEs. A beam-steering device with this multistimuli, multimode capability is presented to show the potential of our material. The initial beam direction can be defined by selecting the temperature, while magnetic fields are independently applied, precisely controlling the beam path around the starting point given by the liquid crystal texture.

## Experimental Section

### Materials

Mesogenic diacrylate (1,4-bis-[4-(6-acryloyloxy-hexyloxy)benzoyloxy]-2-methylbenzene) usually referred to as RM82 or C6M was purchased from Synthon. The photoinitiator (2-benzyl-2-(dimethylamino)-1-(4-morpholin-4-ylphenyl) butan-1-one), commercially known as Irgacure 369 (IRG369), was purchased from Sigma-Aldrich. The chain extender *n*-butylamine and solvent tetrahydrofuran (THF) were also purchased from Sigma-Aldrich. Magnetic particles were obtained from Magnequench GmbH and used as received. The printing substrate was spin-coated with PVA (poly(vinyl alcohol), 80% hydrolyzed; MW between 9000 and 10,000) that was obtained from Sigma-Aldrich.

### Ink Preparation

The magnetically responsive ink was prepared by adding *n*-butylamine and RM82 in a molar ratio of 1:1.2, respectively. Then, IRG369 was added at 2–3.5 wt % depending on the weight percentage of magnetic particles. The percentage of MMPs was within the range of 10–50 wt %. These were added along with the rest of the reactants and an excess of THF. Quickly after solvent addition, the container was sealed and placed under sonication for 10 to 40 min at RT. Following this step, the mixture was heated at 70 °C and stirred at 720 rpm with the flask open until the solvent was completely evaporated. The mixture was further degassed using a negative pressure of 800–900 mbar at 70 °C for 2 h, and after this process, it was transferred into the syringe prior to printing. This mixture becomes the ink used to print the magnetically active LCE.

### 3D Printing Setup

The printing of magnetic actuators was carried out using a home-built 3D printer and heating system for the ink reservoir, as reported in the literature.^23^ The process starts by designing the actuator path-by-path through computer-aided design (CAD) using Libre-Cad freeware, which later was translated into G-code using an in-house coded program, allowing for the fine control over the printing path via WinPC-NC software.

### 3D Printing of Magnetically Responsive LCE Actuators

The ink-loaded syringe equipped with a needle tip (23 gauge with an inner diameter of 330 μm) was placed in the heated reservoir at 70–78 °C, depending on the solid content, and warmed up for 20 min before printing. The LCE elements were printed on conventional microscope glass slides kept at RT. Prior to printing, the glass slides were cleaned, spin-coated with a solution of PVA (5 wt %), and dried at 60 °C for 60 min. After they cooled to RT, they were used for printing. The printing process was dependent on printing speed, extrusion pressure, and ink temperature. Printing was usually done at speeds of 4–22 mm/s and 2–7 bar, the ideal parameters were dictated by the solid content and pattern to be printed.

The geometries used for the printed elements were designed and printed in-house. Immediately after printing the elements, they are photopolymerized by exposure to UV light of 365 nm wavelength from a light-emitting diode (LED) by Thorlabs. The curing process is carried out at RT and for 12 min on each side, at a power of 25 mW/cm^2^. Additionally, when printing multilayer elements, the sample is irradiated for 2 min in between layers to fixate the alignment of the current layer, protecting it from being disturbed by the following ones.

The printed MLCE actuators were separated from the substrate by immersing them in water, where the PVA coating dissolved within a few hours, releasing the MLCE elements. Prior to any further experiment, they were fixed to a small piece of Kapton tape to allow them to dry.

### Magnetic Programming of MLCE Actuators

The free-standing elements were fixed between two glass slides, and later they were positioned within the electromagnet cavity according to the desired magnetization direction. Once the sample was fixed, a uniform magnetic field of 1.2 T was applied for 5 min and turned off, and the sample was finally removed.

### Characterization Techniques

#### Differential Scanning Calorimetry

The liquid crystal to isotropic transition temperature (*T*_i_) of the macromers was determined by differential scanning calorimetry (DSC) using a TA DSC Q-2000 instrument at a scanning rate of 10 °C/min, from −50 to 150 °C. The samples (about 3 mg) were prepared under a nitrogen atmosphere and sealed in aluminum pans. The liquid crystal to isotropic transition temperature was read at the maximum of the corresponding peaks.

#### Polarized Optical Microscopy

LC-printed elements were observed under a polarized optical microscope, Nikon Eclipse 80i.

#### Thickness Measurement

The thickness of the printed and cured elements was measured using a profilometer, Bruker DektakXT Stylus Profiler.

#### Mechanical Characterization

The uniaxially aligned printed elements at 0 and 50 wt % (3 × 20 × 0.1 mm^3^) were mechanically characterized via dynamic mechanical analysis (DMA) using a TA Instruments Q800 equipment with a temperature ramp of 5 °C/min, from 25 to 150 °C. The measurements were carried out in vertical tension mode parallel to the director. From these, their storage modulus (*E*′) was obtained.

#### Magnetic Characterization

The magnetic properties of the printed MLCE were characterized using a Quantum Design PPMS 9T, with a vibrating sample magnetomer (VSM). The samples were printed to size or cut to fit in the sample holder with the printing direction perpendicular to the applied magnetic field coming from the equipment.

### Thermal Actuation

The thermomechanical tests under load were performed in an aluminum oven with a cavity that allows for optical access, enabling the recording of the deformation. The sample was attached to a holder from one end, and a weight of 1 g was hung from the other one. The sample was placed in the cavity while it is at room temperature and gradually heated to 150 °C with a 2.5 °C/min slope. The thermal actuation data was recorded with a digital camera, Nikon D3300. The processing of the data includes measurement of the length and width of the actuator using image analysis software. Shortly, a line was drawn over a physical reference of 1 cm present in the oven to obtain the scale used for all images. Then, a graphic line was drawn starting at the end of the fixation tape to the other one, where the weight was hanging freely. Then, the length and width were normalized with respect to the initial dimensions at RT.

### Magnetic Actuation

The response of the actuators to the magnetic field was quantified using image analysis software Fiji. The images were taken using a digital camera, Nikon D3300. For the bending response, a horizontal line corresponding to the initial resting state of the actuator was drawn. Next, a reference length was defined as 10% in length on the free end of the actuator, where a line tangent to this area was defined. The angle between the horizontal and this line was taken as a measurement of the bending deformation for each magnetic field.

To measure the magnetically induced twisting in the sample, the apparent width (AW) measured as the width projected into the YZ plane was taken as a reference to calculate the angle. The twisting angle was calculated through the arccosine of AW over the real width (*W*) of the printed actuator.

To quantify the curling mode, a line describing the width of the free end was drawn at 0 mT and taken as a horizontal reference. At every increment of magnetic field, a new line was described, and the angle between it and the horizontal reference.

### Multistimuli Experiments: Temperature and magnetic field

For the multistimuli experiments, a custom-made aluminum cavity was heated on a hot plate (Stuart SD160), and then the temperature is incrementally adjusted from RT to 110 °C, in intervals of 5 °C to allow the system to stabilize for 5 min. In the studies of multimodal response of MLCE using magnetic and thermal stimuli, the experiments were carried out at 60 °C and magnetic fields were generated with a simple pair of coaxially aligned and identical coils positioned one at each of the sides of the cavity, with the sample centered between the cores of both coils. Each coil consists of 1000 wire turns and controlled with a single channel from a waveform generator (Tenma Model 75-3555) supplying a 10 V peak (*V*_p_) on each channel.

For the MLCE-based beam-steering device, the images were captured with the device at a temperature of 95 °C and a mirror angle of 94°. The laser beam was projected onto a screen at a specific distance, filling the shown laser paths with a 10 × 10 cm^2^ area. However, these laser paths could be generated in larger or smaller sizes by controlling the distance to the screen. Different actuation temperatures would also lead to different mirror angles and therefore different positions of the laser beam at zero magnetic field. The magnetic field was generated using the previously described pair of coils and a waveform generator. In this experiment, the coils were positioned to have their centers orthogonal to each other. To achieve this, the coils were assembled on top of an optical table, and the field was measured with a gaussmeter (PCE Instruments MFM-3000) at the mirror surface region, once at the desired curvature of the device and thermally stabilized.

### Fabrication of the Beam-Steering Device

The fabrication of the beam-steering device started with the printing and magnetization of a bimorph actuator (7 × 3 mm^2^) using 50 wt % ink, as previously described. Once free, a reflective glass (160 μm thick) was cut manually to fit the width of the actuator (3 × 3 mm^2^) and attached using a thin glue film. Then, the mirror-integrated bimorph was fixed in a nylon clamp to avoid direct contact with the aluminum cavity, ensuring a more homogeneous heating of the sample. The whole device was fixed inside the chamber and heated to the desired mirror angle.

## Results and Discussion

### Formulation of Magnetically Responsive LC Ink, Printing, and Magnetic Programming

**Figure 1:**
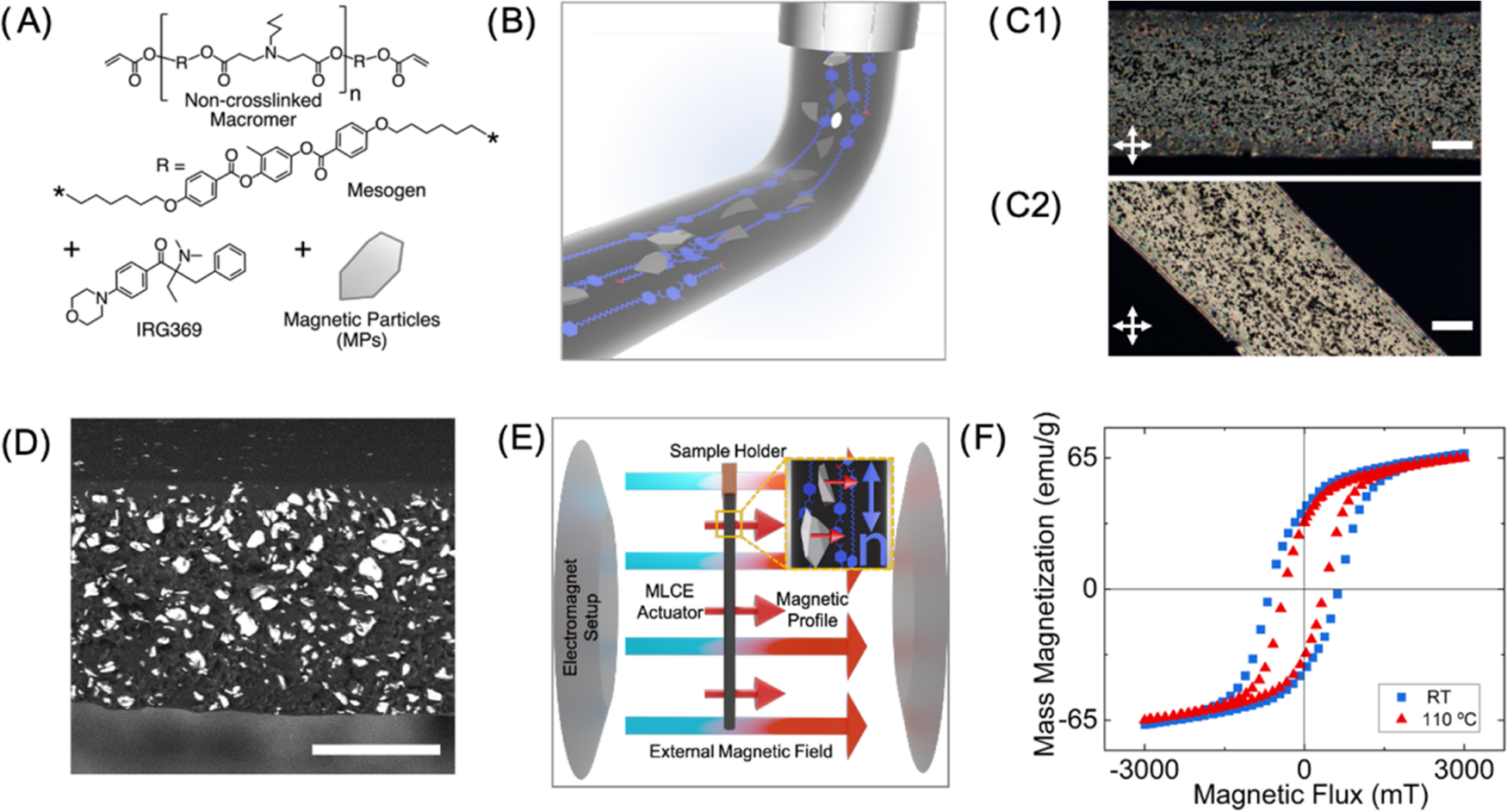
Formulation, processing, and characterization of MLCEs: (A) Components of the printable ink. (B) Schematic representation of the printing process, depicting LCE chains in blue and magnetic microparticles (MMPs) in gray (not to scale). (C) POM images of a 10 wt % solids uniaxial actuator at (C1) 0° and (C2) 45° with respect to the first polarizer transmission direction (horizontal in the pictures) (scale bar: 100 μm). (D) SEM micrograph of a freeze-fractured uniaxial actuator 50 wt % solids, image shown in backscattered electron mode to highlight the particle content (scale bar: 50 μm). (E) Schematic illustration of the magnetization process of an MLCE actuator in an electromagnet setup, magnetizing field *B* in large blue-red arrows, the magnetic moment induced in the material (in particular, in each MMP in the yellow square inset) in small red arrows, and the LCE chains and the director field *n* in blue. (F) Hysteresis curves measured at RT (blue squares) and at 110 °C (red triangles) of a 50 wt % solids, MLCE composite.

Our final MLCE is composed of a mechanically active matrix of LC cross-linked polymer embedding neodymium–iron-boron (NdFeB) hard MMPs. These MMPs, with a flake-shape morphology,^52^ are capable of retaining a magnetization even in the presence of a moderate external magnetic flux, due to their high remanence,^53^ enabling the programming of a magnetization profile (*vide infra*). The printable precursor material for the final MLCE is composed of an acrylate-ended main chain LC macromer and an ultraviolet (UV) photoinitiator (IRG369), together with the nonmagnetized particles, at a solid content between 0 and 50 wt % (Figure 1A). To obtain the reactive macromer, the chain extender *n*-butylamine is reacted with C6M via the Aza-Michael reaction.^35^ A slight deficit of *n*-butylamine compared to the diacrylate content results in a non-cross-linked macromer with a sufficiently high polymerization degree. The pure macromer displays thermotropic behavior and a *T*_i_ at 104 °C upon heating, according to our DSC measurements and polarization optical microscope (POM) observations. The addition of MMPs to the macromer led to a small decrease of the *T*_i_ with increasing solid contents, with a *T*_i_ of 99 °C for the sample with 50 wt % MMPs (Table S1 in the Supporting Information). The macromers can be easily extruded into fibers while retaining acrylate functionalities at both chain ends. The presence of the photoinitiator facilitates UV-initiated free-radical polymerization of these reactive acrylate groups, making the LC macromers turn into the final LCE scaffold.

Then, this mixture is used as ink in a 3D printing home-built platform provided with a heated extruding nozzle.^23^ The direction, speed, and extruding rate are dictated by computer numerical control (CNC) via custom-made g-code, generated from a CAD drawing. It is known that shear and elongational forces during filament extrusion and deposition in main chain LC polymers can lead to alignment of mesogens parallel to the movement direction of the nozzle.^23,37,40,42^ In this manner, the LC alignment can be digitally defined during the printing process (Figure 1B). This is also seen for the composite precursor system including MMPs, Figure 1C shows polarized optical microscope (POM) images at 0° (Figure 1C1) and 45° (Figure 1C2) with respect to the first polarizer transmission direction, for a 10 wt % MPs content uniaxial actuator. The POM observations are compatible with a mesogen orientation parallel to the printing direction, as previously described in the literature.^23,37,40,42^ Once the material is extruded and deposited, the printed element is UV-exposed to activate the photoinitiator and cross-link the free acrylate groups at the chain ends leading to LCE elements. The alignment imparted during processing is retained after the curing process as checked by POM (Figure 1C) even if photoexposure is carried out tens of minutes after deposition. The resulting LCEs present a gel fraction between 93 and 97% of insoluble material, indicating an efficient light-induced polymerization process.

Notably, the precursor material holds its good processability and leads to printed elements at higher solid contents such as 25 and 50 wt %, with a well-defined director, that is ascribed to the good dispersion of MMPs within the LC ink. This dispersion and embedment of magnetic particles within the cured LCE is confirmed by observing a scanning electron microscope (SEM) image of the morphology present in a 50 wt % uniaxial printed element, shown in Figure 1D. The micrograph, which shows the cross section of the freeze-fractured actuator, was taken in backscattering mode, to highlight the well-distributed solid content along the whole volume of the sample. Using this imaging mode, the particles show a brighter contrast due to the higher atomic mass of the MMPs,^54^ compared to those in the LCE matrix appearing in darker tones. Despite the flake morphology of the MMPs, no alignment of these has been observed under the used extrusion conditions. It is worth noting, regarding the stability of the inks, that these remained homogeneous and printable with similar printing conditions for more than 6 weeks at any of the studied solid contents.

The inclusion of MMPs allows the possibility of programming the particles’ magnetic moment in defined directions using a strong external magnetic field *B* (1.2 T). This is schematically presented in Figure 1E, in which the blue-red arrows represent the external magnetizing field, and the small red arrows represent the magnetic moment induced in the materials and, in particular, each MMP, as shown in the yellow square inset. In the example presented in Figure 1E, the magnetic moment is perpendicular to director *n* (represented in the *inset* as a blue double-headed arrow), endowing the material with magnetic response capabilities.

To ensure the actuator retained its magnetic properties after programming, even in the presence of the thermal stimuli needed to activate its LC response, hysteresis curves of the MMP (Figure 1F) were measured at room temperature (RT) and 110 °C, where the expected loss in magnetization is less than 15%.^53^ The measurements performed using a vibrating sample magnetometer (VSM), exhibited a high remanence and strong coercivity despite the high temperatures, demonstrating the retention of the magnetic programming, potentially leading to a strong magnetic response even to relatively low magnetic fields.

To summarize, the inclusion of MMPs in a printable ink allows the possibility of two orthogonally programmable properties within the same material. On one side, the alignment of the director field in the LCE matrix that is imparted during the printing process (Figure 1B), and on the other side, the programming of the particles’ magnetic moment in defined directions using a strong external magnetic field (Figure 1E).

### Thermomechanical Response of MLCE Actuators

**Figure 2:**
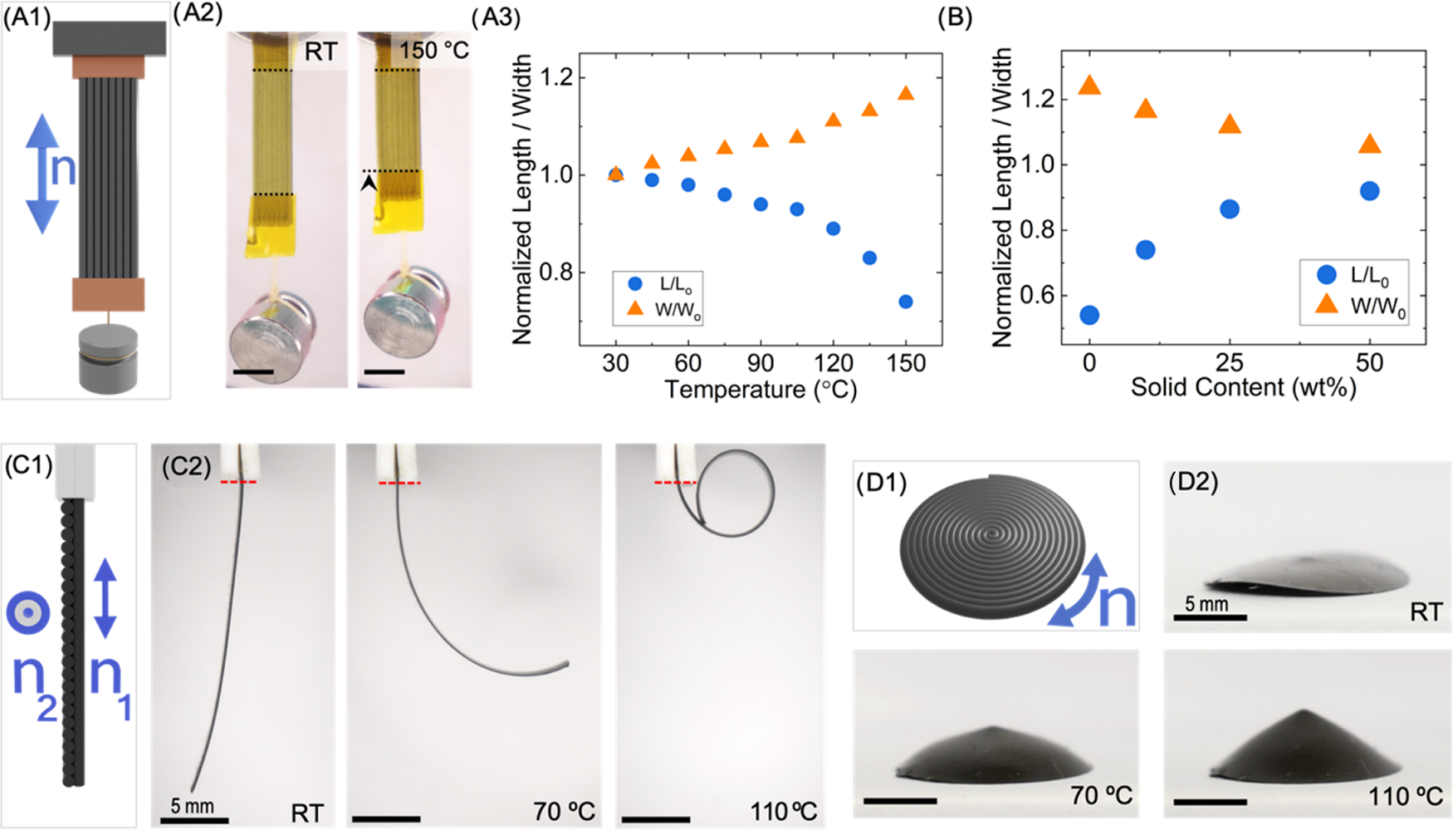
Thermomechanical actuation of MLCE elements. (A) Thermomechanical response of a uniaxially aligned MLCE sample. (A1) Schematic view of a thermomechanical test, a printed element with director *n* (indicated as a blue double-headed arrow) parallel to the long axis of the sample, is fixed from one of its ends (top), then a load of 1g is attached to the freely hanging end (bottom). Then, heat is applied using an oven with optical access, starting at RT and reaching 150 °C. (A2) Thermomechanical test of a 10 wt % MPs LCE sample (110 μm thick), uniaxially oriented as depicted in (A1). Pictures were taken at RT and 150 °C. Black dashed lines in these pictures indicate the fixation tape endings used as a reference to measure the length of the actuator (*L*) as a function of temperature (scale bar: 3 mm). (A3) Change in normalized length (*L*/*L*_0_) (blue circles) and normalized width (*W*/*W*_0_) (orange triangles), for a 10 wt % actuator during the thermomechanical test. Width (*W*) is measured at the middle of the actuator sample as a function of temperature. The normalization is done using the length and width values at RT, *L*_0_, and *W*_0_, respectively. (B) Thermomechanical actuation at 150 °C and load of 1 g for 0, 10, 25, and 50 wt % MMPs, uniaxial actuators, showing the change in normalized length (blue circles) and width (orange triangles) as a function of solid content. (C, D) Thermally actuated elements of LCE with 50 wt % MMPs, with different director patterns: (C1) Schematic representation of a bilayer element showing the cross section with the two printed layers. Layer 1 with a director *n*_1_ parallel to the long axis of the stripe (vertical in the image), and layer 2 with a director *n*_2_ along the short axis (perpendicular to the image) The bilayer element is hung from one of its ends. (C2) The element shown in (C1), with the same described orientation of layers, progressively bends on heating from RT, closing in a loop at 110 °C (scale bar: 5 mm). (D1) Schematic representation of a disk having a spiral-like director texture *n*. (D2) The actuator, with this director pattern, presents a slight saddle-like shape at RT and forms a conical shape when thermally actuated at 110 °C (scale bar: 5 mm).

Prior to performing magnetic actuation experiments in our printed MLCE elements, temperature-induced deformation has been assessed. First, we explore the thermomechanical actuation of one-layer uniaxial printed MLCE actuators at different solid contents (Figure S1). The experiment was performed as schematically seen in Figure 2A1 with the molecular alignment direction *n* parallel to the long axis of the element, the same as the printing direction. The actuators are placed in a sample holder fixing one end to it, then, a 1g load is attached to the hanging end, and later, the sample is placed inside an oven for the heating process. The oven has an optical access that allows to observe the samples during heating starting at RT and ending at 150 °C. Figure 2A2 shows the images of the sample at these temperatures for the case of a 10 wt % MMPs LCE. This test allows quantification (Figure 2A3) of the contraction in the sample’s length (*L*, indicated by blue circles) and the increase in width (*W*, indicated by orange triangles) of the printed element. Both of these quantities are measured and then normalized to the initial dimensions (*L*_0_, *W*_0_) that were measured at RT. These dimensional changes are ascribed to the thermally induced increase in mesogenic disorder, as previously described for LC cross-linked systems.^21,27^ The 10 wt % MMPs LCE sample presents at 150 °C a contraction of 26% of its initial length and a normalized expansion of 17% along its short axis, a deformation that is reversible when cooling down to RT. Figure 2B shows the length (blue circles) and width (orange triangles) at the highest temperature studied of 150 °C for uniaxial elements of different MMPs content, ranging from 0 up to 50 wt % solid content. These length and width are displayed normalized to the corresponding dimensions at RT. In addition to the already described 10 wt % MMPs LCE results, the actuators with higher solid content exhibited thermal responses, resulting in a 14% contraction in length and a 12% expansion in width for the 25 wt % MMPs sample in the same temperature range. Similarly, an 8% contraction in length and 6% expansion in width was measured for the 50 wt % MMPs elastomer (Figure S2). Besides, the reference LCE without MMPs shows a 46% contraction in length and a 24% expansion in width. Therefore, the actuators fabricated with MLCE composite can lift the 1g load to a significant height in all of the cases, regardless of the MMPs content. It is important to note that it was checked that for all of the studied actuators, the 1 g load does not compromise the performance of the actuator, finding comparable thermomechanical contraction with unloaded samples within the experimental error (see Figures S3 and S4).

It is possible to understand that the lower changes in dimensions as the solid content increases might be caused by the increasing particle volume that does not contribute to thermally induced deformation and is incompressible under these conditions. However, it cannot be disregarded that achieving a relevant, but lower molecular alignment in the higher percentage range of solids may also lead to a lower mechanical response. The storage modulus (*E*′), measured using DMA, has also been characterized for uniaxial actuators at 0 and 50 wt %, as shown in Figure S5 in the Supporting Information. The experimental findings indicate that the inclusion of MMPs in the formulations leads to an MLCE with a higher storage modulus across the measured temperature range.

The 50 wt % MMPs ink has been chosen as the primary formulation to further explore and characterize the presented material and method. This choice is motivated by the significant thermomechanical response exhibited by the resulting MLCE, along with its higher magnetic mass content, which is anticipated to enhance its response to a magnetic field.

Different director patterns and elements were printed (Figure 2C,D) to further explore the possibilities to program the director configuration in LCEs with a high MMPs content. The design for a bilayer long element is illustrated in (Figure 2C1), where the first layer of the element is printed with lines parallel to the long axis, and those in the second layer are in the perpendicular direction with respect to the first one. The resulting director is denoted by *n*_1_ and *n*_2_, respectively, in Figure 2C1. The printed element (Figure 2C2) with dimensions of 30 × 5 mm^2^ (*L* × *W*) and a thickness of 190 μm is freely hanging fixed from one end. The sample, that is nearly flat at RT, increases its curvature as the temperature rises, finally completing a loop at 110 °C. By exposing the actuator to heat, and due to the subsequent molecular disorder, the first layer, printed with lines parallel to the long axis of the bilayer element, tends to contract along this direction. Concurrently the second layer, printed with lines parallel to the short axis, tends to expand along the same long axis of the element. As a result of these heat-triggered stresses within the long strip, this bends outside the plane and finally forms the loop (Movie S1).

Also, complex patterns in the plane can be imparted to the MLCE using 4D printing, as demonstrated in (Figure 2D) where a spiral-like texture described in (Figure 2D1), is printed as a disk measuring 12 mm in diameter and a thickness of 95 μm. This spiral pattern is an approximation of an azimuthal director field around a + 1 disclination, but with a continuous printing track that leads to good quality elements with our printing platform. The sample exhibiting a slight saddle-like shape at RT (Figure 2D2) is thermally actuated. As temperature increases the material starts developing a rising apex at the center of the spiral as theoretically predicted by Warner and co-workers for azimuthal director samples.^55^ The cone takes a more defined shape as the apex continues to move upward, sharpening the cone at 110 °C (Movie S2). The thermally induced increase of molecular disorder induces a contraction along the spiral direction and concurrently an expansion along the radial one. The system can accommodate these stresses by morphing into a cone.

Overall, the thermally induced response of these printed MLCE elements confirms the ability to impart the complex morphing characteristic of LCE matrices using 4D printing, even with a particle content increased up to 50 wt % of MMPs.

### Magnetic Actuation of MLCE Elements

**Figure 3:**
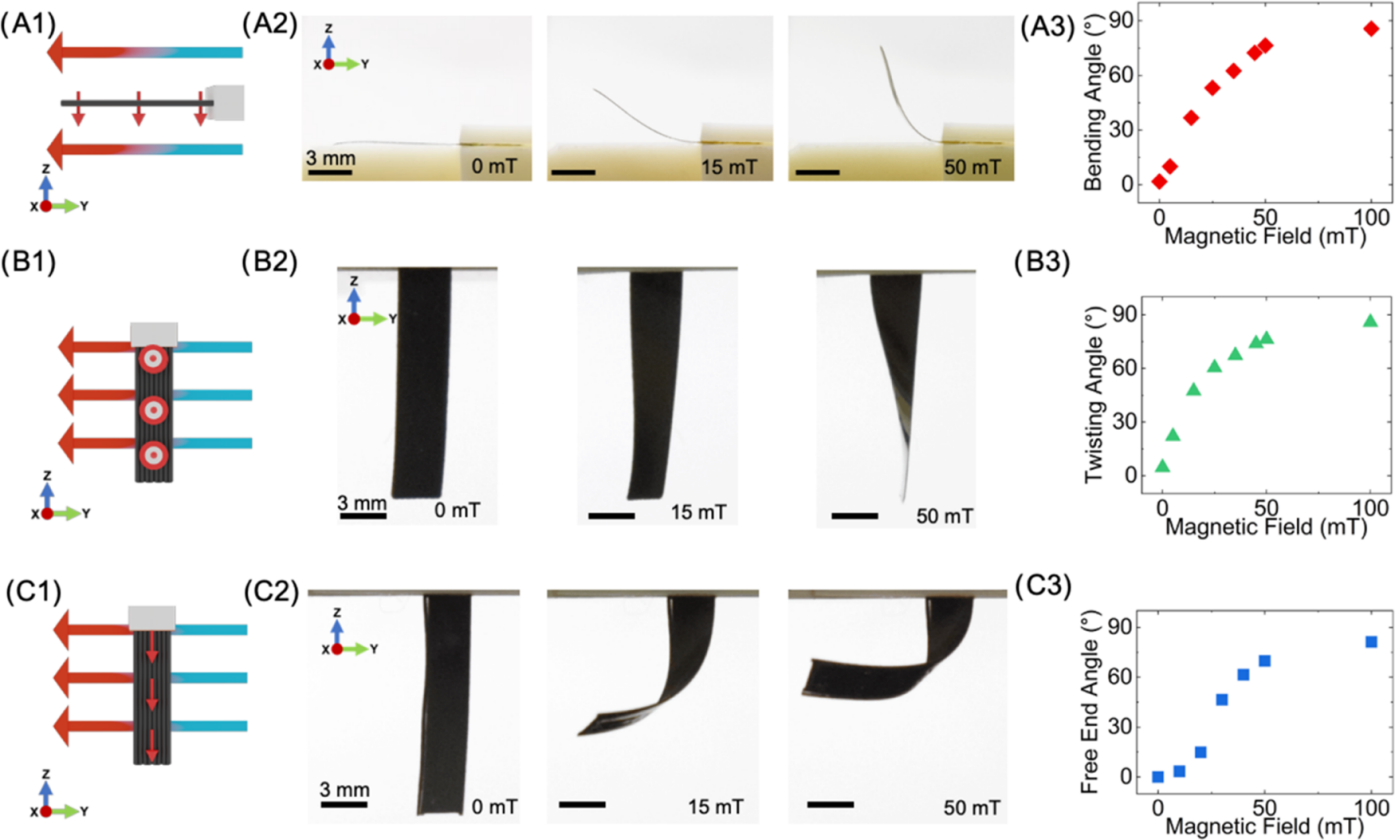
Magnetic actuation modes. An external magnetic field (large blue-red arrows in the schematic representations) was applied on the printed samples (shown in black), having these defined magnetization directions (small red arrows). The samples, measuring 15 × 3 mm^2^ (*L* × *W*) with a thickness of 80–90 μm and 50 wt % MMPs content, are fixated by one of its ends (white block). Three different experimental configurations have been studied, and the deformation quantified through image analysis. (A) Bending deformation. (A1) Schematic representation of the experimental configuration for a sample previously magnetized in the direction perpendicular to its surface. The sample is initially positioned with its magnetic moment perpendicularly entering into the XY plane at a zero magnetic field. (A2) Images at increasing magnetic field showing sample bending. (A3) Tip bending angle with respect to the horizontal (−*Y* axis) as a function of the applied magnetic field. (B) Twisting deformation. (B1) Schematic representation of the experimental configuration for a sample previously magnetized in the direction perpendicular to its surface. The sample is initially positioned on the YZ plane at a zero magnetic field. (B2) Images at increasing magnetic field showing sample twisting. (B3) Twisting along the long axis of the sample as a function of the applied magnetic field, characterized by measuring the angle of the free end of the sample with the *Y* axis. (C) Curling deformation. (C1) Schematic representation of the experimental configuration for a sample previously magnetized along its long axis and its magnetic moment positioned perpendicularly entering the XY plane at zero magnetic field. The sample is initially positioned on the YZ plane with no field applied. (C2) Images at increasing magnetic field showing sample curling. (C3) Angle that defines the free end, projected into the YZ plane with the *Y* axis.

Once the capability of our LCE material to be programmed via 4D printing has been assessed, the use of magnetic fields is explored to integrate a magnetic response into the actuator. MLCE rectangular actuators (15 × 3 mm^2^*L* × *W*, 80–90 μm thick) with uniaxially oriented director along their length, were magnetized in defined directions (small red arrows) (Figure 3), employing a uniform magnetic field of 1.2 T. The samples are fixed at one extreme in different orientations remaining flat at RT when no magnetic field is applied. A relatively small magnetic field, up to 100 mT (blue-red arrows in Figure 3), is applied to the samples using the same electromagnet setup schematically depicted in Figure 1E. Due to the magnetic moment *m* acquired during the magnetization process, these samples, under the action of a magnetic field *B*, locally experience a torque τ as described by the equation
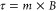
1As a result, we anticipate that our samples, under a homogeneous magnetic field, will tend to deform, aligning the local magnetic moments at the free end of the actuator with the direction of the magnetic field.

In the first configuration, a printed element, previously magnetized with direction perpendicular to its surface, is fixed (Figure 3A1) on its right end to a fixed block (white block in the figure). In the absence of any external magnetic field, the sample surface lies in the XY plane. In this situation, the sample has the magnetic moment perpendicularly coming into this XY plane and pointing downward. To magnetically actuate the sample, a homogeneous magnetic field, depicted as blue-red arrows, is applied. This magnetic field is aligned with the *Y* axis and points toward the left in the figure. As the magnitude of the magnetic field increases, the elements bend upward (Figure 3A2,A3). The torque exerted by the magnetic field on the MMPs within the element increases with the strength of the magnetic field, causing alignment of the local magnetic moments at the free end of the element with the field. Large deformations are achieved for magnetic fields in the order of tens of mT being the angle of the tip close to 90° at 100 mT.

In a second configuration (Figure 3B1), the sample possesses the same magnetization as the just described uniaxially aligned sample. However, in this case, the orientation of the element differs with respect to the actuating magnetic field. In this configuration, the sample hangs from a fixed block (white block in the figure), having its surface, under no magnetic field, lying in the YZ plane. The magnetic moment is perpendicular to the YZ plane, aligned with *X* axis and pointing toward the reader. When a homogeneous magnetic field, aligned with the *Y* axis and pointing toward the left in the figure, is applied, the sample experiences a twisting along its long axis (Figure 3B2) with the twisting angle being larger and approaching the maximum achievable angle of 90° for larger magnetic actuating fields (Figure 3B3). As in the previous case, the magnetic field tends to align the magnetic moments of the free end of the element with the magnetic field.

In the third configuration (Figure 3C1), the sample, as in the previous case, hangs from a fixed block, and its surface lies in the YZ plane, in the absence of a magnetic field. In this case, the sample has been previously magnetized with its magnetic moment parallel to the long axis of the sample and pointing downward in the figure. In this case, the same external actuating field configuration is employed (aligned with the *Y* axis and toward the left in the figure), as in the previous two configurations. An increase in the magnitude of the magnetic field makes the sample locally curl in such a way that the long axis of the element aligns, at its free end, with the magnetic field (Figure 3C2,C3). Again, this phenomenon can be explained by considering eq 1.

These experiments contribute to the understanding of mechanical deformations solely generated by a magnetic field in the 4D-printed MLCE devices. It was observed that a major fraction of the possible range of actuation can be reached at fields below 50 mT and a fair portion of it below 10 mT, widening the scenarios where these devices could be employed by using low actuating magnetic fields.

### Multimodal Response Enabled by Magnetic and Thermal Actuation

**Figure 4:**
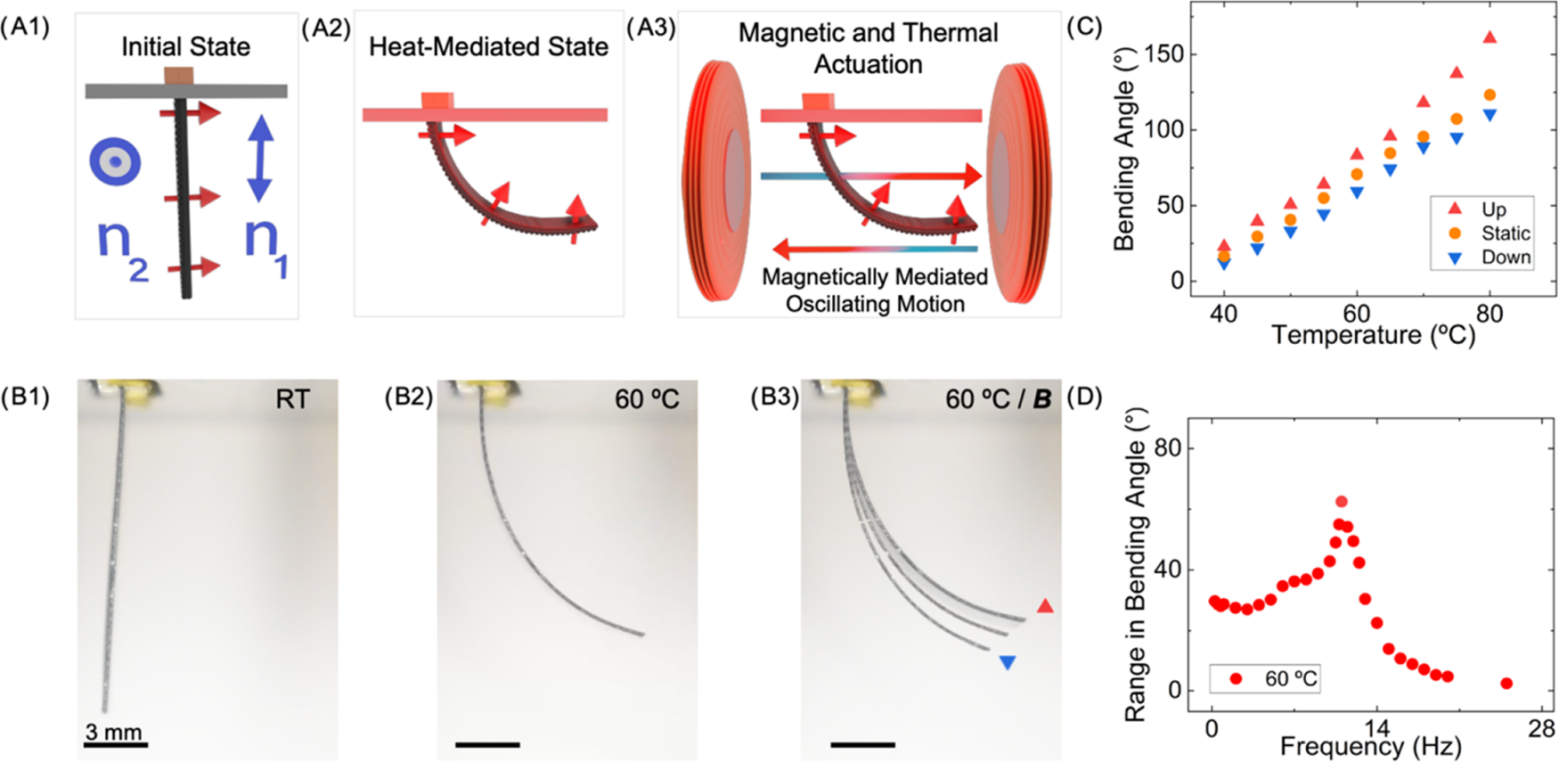
Multimodal response of MLCE using magnetic and thermal stimuli. (A) Schematic representation of the multistimuli, multimodal behavior. The bilayer MLCE actuator has a layer 1 with a director *n*_1_ parallel to the long axis of the stripe (vertical in the image), and layer two director *n*_2_ along the short axis (perpendicular to the image). The sample with dimensions 15 × 3 mm^2^ (*L* × *W*) and 190 μm thick is fixed at the upper end and free at the lower end. (A1) *Initial State*: printed and magnetized element with no stimuli provided. The magnetization profile of the element is perpendicular to its surface (red arrows). (A2) *Thermal Actuation*—*Heat-mediated state:* Heat is applied to reach a defined temperature and the, previously uniform, magnetization profile is now locally curved due to the LCE matrix thermoresponse. (A3) *Magnetic and Thermal Actuation*—*Magnetically mediated oscillating Motion:* At each of the sides of the heating cavity, a pair of coils applies a sinusoidal magnetic field of ±10 mT peak at the location of the actuator, at a frequency of 5 Hz. The magnetic input makes the actuator respond in an oscillating motion around the heat-mediated state. (B) Multistimuli, multimodal behavior of an MLCE-printed actuator as depicted in (A). (B1) Bilayer MLCE actuator (50 wt % MMPs) at RT in the initial state, with the same director configuration and magnetization direction as in (A1). (B2) Static bent state reached 60 °C. (B3) Composition of juxtaposed images showing the oscillation amplitude with upper (red triangle) and lower (blue inverted triangle) positions of the oscillating motion when the sinusoidal magnetic field is applied. (C) Maximum (red triangle) and minimum (inverted blue triangle) tip bending angle as a function of temperature, as observed in (B). The tip bending angle at the static, heat-mediated state at zero magnetic field is represented as an orange dot for each temperature. (D) Range in bending angle of oscillation at a heat-mediated state of 60 °C and different frequencies of the applied magnetic field.

Combining thermal and magnetic stimuli can lead to modes of actuation that are difficult to access by using only one single input. This possibility was explored by using a MLCE bimorph actuator as previously described in Figure 2C with dimensions 15 × 3 mm^2^ (*L* × *W*) and 190 μm thick. The element is printed and magnetically programmed with its magnetic moment perpendicular to its surface. In the absence of magnetic field, the sample, which is fixed at the top end, remains flat as schematically shown in Figure 4A1, (referred to as *Initial state)*. The sample is placed in a homemade temperature-controlled aluminum cavity with optical access. The system is initially at RT with no magnetic stimulus applied (Figure 4B1). Next, the oven cavity is heated to increasingly higher temperatures and the bimorph responds gradually bending. At high temperatures, the bimorph achieves greater curvatures due to the programmed director pattern within the LCE matrix, reaching the *Heat-mediated state* shown in Figure 4A2. As a result of this reconfiguration, the magnetization profile, which was uniform in the initial state at RT, undergoes local rotation in space following the element’s surface shape change, as illustrated in Figure 4A2. Figure 4B2 shows this thermally actuated state of the MLCE bimorph, as observed at 60 °C. This heat-mediated state serves as starting point to magnetically actuate the sample. In particular, a *magnetically mediated oscillating motion* is achieved by applying a small oscillating magnetic field at the sample’s location, as shown in Figure 4A3. This field is directed along the horizontal direction in the figure and alternatively points to the left and to the right within the period of the oscillation, as schematically indicated by the blue-red arrows in Figure 4A3.

In Figure 4B3, a superposition of images highlights the extreme actuation states reached under thermal excitation (*T* = 60 °C) and simultaneous magnetic addressing. The test input was a sinusoidal magnetic field at 5 Hz and a 10 mT peak (see Movie S3). During the positive part of the sine, corresponding to a field pointing from right to left, the bilayer bends upward (Figure 4B3, red triangle). Conversely, during the negative part of the sine, the magnetic field points from left to right, the bilayer bends downward (Figure 4B3, blue triangle). Although the inhomogeneous character of the magnetic field generated by the employed coils could lead to forces due to magnetic field gradients, the observed phenomenon can be understood considering the magnetic torque from eq 1. In this way, with this multistimuli actuation scheme, the LCE matrix morphology dictates a heat-controlled bending angle, around which a magnetically addressed oscillation takes place, this one driven by the magnetization direction and the external magnetic field (see Figure 4C).

The effect of the magnetic field frequency over the oscillation amplitude, as defined by the range in bending angle between the upward and downward motions, is shown in Figure 4D, for the actuator at 60 °C. The shape of the plot follows that of the amplitude as a function of the driving frequency in forced-damped oscillators. In these systems, resonance occurs when the driving frequency matches the natural frequency of the oscillator. Our long stripe, naturally damped by its viscoelastic character, is forced by the oscillating magnetic field, thus leading to this resonant response. From experimental data at 60 °C, it is possible to identify a resonance close to 11 Hz where the amplitude is maximum. For a given oscillation mode, this resonant frequency depends on material properties, such as density and mechanical properties, as well as geometry. As a result, this frequency could be tailored by changing the design parameters or the matrix properties, via formulation or processing.

Summarizing this part, the excitation with multiple stimuli of our MLCE 4D-printed actuators gives access to actuation modes difficult to reach with one single stimulus. For example, as shown, the thermally induced response of 4D-printed elements provides the actuator with a well-defined new state around which the magnetic actuation takes place. Although stated like this, the way the stimuli are applied can be changed leading to a wider variety of complex motions, a highly desirable feature in soft actuators.^9,46^

### Multistimuli, Multimode Actuation for Optical Beam-Steering Devices

**Figure 5:**
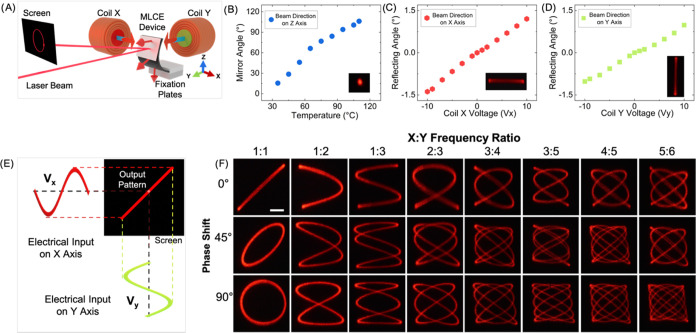
MLCE-based beam-steering device. (A) Schematic representation of the beam-steering device. A bilayer MLCE actuator consists of a long stripe (7 × 3 mm^2^, *L* × *W*) with a total thickness of 130 μm. One of the layers is printed with lines parallel to the long axis of the stripe, and the second layer is printed with lines parallel to the short axis of the element. The sample is magnetically programmed in such a way that the local magnetic moment is perpendicular to the surface (red arrows). The bilayer MLCE actuator is integrated with a thin mirror at one of its ends that is free. The other end of the sample is fixed between two nylon fixation plates. The whole device is positioned inside an aluminum chamber (not shown for clarity purposes) with glass windows. This provides optical access to the sample and homogeneous heating, the same as that that induces bending of the actuator as shown in the figure. Two pairs of coils are positioned outside the chamber to generate a magnetic field in the X direction (Coil X) and the Y direction (Coil Y) at the sample position. A laser beam points to the mirror of the MLCE device. The light reflected in the mirror is projected onto a screen. (B) Upon heating, the bilayer increases its curvature, bending the mirror-integrated actuator’s end upward, in a wide range of angles with respect to the horizontal, reflecting the beam at such angles (Figure 5B, inset). (C) Coil X (shown in Figure 5A with a red core) generates a magnetic field (indicated as a blue-red arrow close to the coil) that tends to produce a torsion at the actuator’s end and, thus, steer the beam horizontally (Figure 5C, inset; picture is taken using long exposure photography to capture the beam path). (D) Coil Y (shown in Figure 5A with a green center core) generates a magnetic field (blue-red arrow) that tends to produce a bending of the actuator’s end and thus leads to deflection of the beam in the vertical direction (Figure 5D, inset; picture using long exposure photography). (E) Schematic representation of the creation of Lissajous patterns. These are formed by applying two sinusoidal in-phase electrical signals, *V*_*x*_ and *V*_*y*_, of the same frequency to the coils. The coils respond to these signals, producing sinusoidal and in-phase magnetic field inputs. That will result in a beam linear trajectory at 45° reflected on the screen. (F) Pictures of the beam traces using long exposure photography, as produced by the steering device, when sinusoidal signals are applied in both coils, using a set of X:Y frequency ratios and phase shifts that render a concise demonstration of Lissajous patterns (scale bar 3 cm), MLCE actuator is at a temperature of 95 °C.

As an application example of the MLCE platform and taking advantage of the just described multistimuli, multimode capability, a beam-steering device, schematically shown in Figure 5A, was developed. The fabrication of the device started with the printing of a bilayer actuator with dimensions of 7 × 3 mm^2^ (*L* × *W*) and a thickness of 130 μm. One of the layers is printed leading to a director parallel to the long axis of the stripe and the other layer along the short axis. The sample is magnetized perpendicular to its surface, as in the previous section. A small reflective glass (3 × 3 × 0.16 mm^3^, 3.2 mg) is integrated on the actuator’s surface at which the magnetic moment (indicated with small red arrows) points out, as shown in Figure 5A. The other end of the bilayer is fixed in between two nylon plates, as to leave, at RT, the actuator nearly lying in the XY plane with the long actuator axis aligned in the Y direction and the mirror facing downward. The orientation of the director in the bilayer is such that heating of the actuator will induce its bending up so the mirror is upright positioned, as schematically shown in the figure. As in the previous set of experiments, our MCLE beam-steering device is inside a temperature-controlled chamber with optical access. A Helium–Neon (HeNe) laser beam, emitting light at 632.8 nm, is directed through the chamber optical aperture, parallel to *Y* axis, to eventually reach the mirror-integrated MLCE bilayer device, once this is thermally actuated (*vide infra*).

Heating the actuator from RT to a higher temperature results in a progressive upward bending of the sample, consequently increasing the angle of the mirror and with it the direction of the beam on the *Z* axis, as shown in Figure 5B. This temperature-driven transformation allows for the coverage of a wide range of angles, up to 110°, with a simple heat stimulus.

Outside the chamber, two coils are placed in a 90° angle configuration, with the sample positioned at the crossing of the coil cores’ axes, as depicted in Figure 5A. When an electrical current is injected into these coils, they generate a magnetic field that enables the magnetic actuation of the bilayer, as previously demonstrated in Figure 3. We have checked that the magnetic field generated follows a linear dependence with input voltage, as shown in Figure S6. On the one hand, the application of a current in coil X (with a red central core in Figure 5A) induces a magnetic field along the *X* axis leading to actuator torsion. This deformation leads to a horizontal beam deflection (Figure 5C, Movie S4) that has a nearly linear dependence on the magnetic field. On the other hand, the application of a current in coil Y (with a green central core in Figure 5A) induces a magnetic field along the *Y* axis leading to actuator bending. This bending leads to vertical beam deflection (Figure 5D, Movie S4) also with a nearly linear dependence on the magnetic field. As a result of this magnetic actuation, varying the input electrical signal applied to both coils enables the dynamic positioning of the beam in the screen. In our experimental setup, we can generate magnetic fields in the order of a few mT in the region of the beam-steering device. This results in a range of deflection angles spanning several degrees, as shown in Figure 5B–D.

Given the nearly proportional relationship between deflection angle, with respect to the applied magnetic field in both axes, and the linear response of the magnetic field generated by the voltage input (Figure S6) for our experimental conditions, we anticipate that a driving sinusoidal waveform in one of the coils will lead to a deflection angle described by a sinusoidal function of time. To demonstrate the high degree of control of the beam deflection of our system in both axes, we have explored the formation of Lissajous curves on the screen using the beam-steering device. The process to generate these curves with the MLCE device is illustrated in Figure 5E. The system is heated at the desired temperature in the range from 35 to 115 °C, for example, at 95 °C, that will define the curvature of the MLCE-based device and thus the reflecting angle, at zero magnetic field, of the incoming beam. At temperatures closer to RT this angle will be lower, with respect to the horizontal, than at higher temperatures, as observed previously (Figure 5B). Then, at 95 °C, a sinusoidal voltage input on coil X (1 Hz) is applied in phase with an equal waveform on coil Y (1 Hz). As there is no phase shift (0°) and the frequency ratio is 1:1 (X:Y), and given that the voltage amplitude of each coil has been adjusted to reach an identical maximum beam deflection in both axes, the beam travels at the same amplitude, phase and frequency on both the *X* and *Y* axes. This results in the beam’s path forming a line at a 45° angle on the screen, as photographed in (Figure 5F, [0, 1:1] - [phase shift, X:Y frequency ratio]).

The ratio of the frequencies between X and Y inputs, as well as the phase difference between them, defines the variety of trajectories created by the reflected beam, as shown in Figure 5F. There, a noncomprehensive set of patterns corresponding to various frequency ratios and the phase shift values of 0, 45, and 90° is displayed, as generated by the beam-steering device. This demonstrates the precise response of the device to magnetic stimuli, highlighting its potential application in the field of optical and sensing devices.

## Conclusions

In this work, we present a new composite ink, prepared from LC macromers and MMPs, that is deposited by using 3D printing. This extrusion-based method allows a well-defined orientation of the mesogenic units achieved through rheological means, enabling the generation of complex director textures, even in thick and large elements and at high concentrations of MMPs. These characteristics tackle a significant limitation of liquid crystal polymer thin-film technology, which is the restricted actuation energy available in thin films.

The precisely defined director morphology, established via 4D printing, enables the demonstration of a versatile multimodal actuator, responsive to magnetic fields. By applying heat, we achieve the reconfiguration of the actuator into multiple states that are subsequently controllable with precision and speed using magnetic fields. For instance, in scenarios where reconfigurable, multimodal fast-actuating devices are required, such as within confined spaces like the human body, provided they are properly adapted to such an environment. These enhanced functionalities may unlock further opportunities in mechanobiology, microfluidics, sensing, and soft robotic applications. To exemplify its potential, an application in the field of optics is presented here through the implementation of a beam-steering device. The initial direction of the beam is defined by thermally actuating the LCE, which will increase its curvature and with it the reflecting angle. Magnetic fields can then be independently applied to precisely control the beam’s direction around that initial state. In the future, assisted by its additive character, the possibility to be automated, and its scalable nature, 4D printing could open the door for new applications in MLCEs. For example, the system could be implemented in an array of devices for multibeam steering or scaled down as needed, potentially being useful in portable scanning applications.

## Supporting Information Available

Liquid crystal to isotropic transition temperature (*T*_i_) of the macromers including MMPs at different solid contents (Table S1); photograph of free-standing uniaxial actuators printed at different solid contents (Figure S1); thermomechanical test of a 50 wt % MMPs uniaxial actuator (Figure S2); thermomechanical response of uniaxial actuator (85 μm) at 0 wt % MMPs with 1 g load and load free (Figure S3); thermomechanical response of uniaxial actuator (110 μm) at 10 wt % MMPs with 1 g load and load free (Figure S4); DMA storage modulus (*E*′) of LCE uniaxial actuator at 0 wt % MMPs (105 μm) and 50 wt % MMP uniaxial MLCE actuator (110 μm) (Figure S5); magnetic field generated by the coils used in Figures 4 and 5, measured at different distances from the surface of the coil (Figure S6); and experimental conditions for thermal and multistimuli tests (Supplementary Note 1) (PDF)
Thermal actuation, of a magnetically responsive liquid crystal elastomer, 4D-printed element bilayer (Movie S1) (MP4)
Thermal actuation, of a magnetically responsive liquid crystal elastomer, 4D-printed spiral-like element (Movie S2) (MP4)
Magnetic and thermal actuation of a bilayer actuator of magnetically responsive liquid crystal elastomers (Movie S3) (MP4)
Multistimuli, multimode beam-steering device directing a beam on the *XY* axis and examples of Lissajous patterns (Movie S4) (MP4)

## References

[ref1] Abbott J. J.; Diller E.; Petruska A. J. Magnetic Methods in Robotics. Annu. Rev. Control Robot. Auton. Syst. 2020, 3 (1), 57–90. 10.1146/annurev-control-081219-082713.

[ref2] Kim Y.; Parada G. A.; Liu S.; Zhao X. Ferromagnetic Soft Continuum Robots. Sci. Robot. 2019, 4 (33), eaax7329 10.1126/scirobotics.aax7329. 33137788

[ref3] Kim Y.; Yuk H.; Zhao R.; Chester S. A.; Zhao X. Printing Ferromagnetic Domains for Untethered Fast-Transforming Soft Materials. Nature 2018, 558 (7709), 274–279. 10.1038/s41586-018-0185-0. 29899476

[ref4] Wu S.; Ze Q.; Zhang R.; Hu N.; Cheng Y.; Yang F.; Zhao R. Symmetry-Breaking Actuation Mechanism for Soft Robotics and Active Metamaterials. ACS Appl. Mater. Interfaces 2019, 11 (44), 41649–41658. 10.1021/acsami.9b13840. 31578851

[ref5] Ze Q.; Kuang X.; Wu S.; Wong J.; Montgomery S. M.; Zhang R.; Kovitz J. M.; Yang F.; Qi H. J.; Zhao R. Magnetic Shape Memory Polymers with Integrated Multifunctional Shape Manipulation. Adv. Mater. 2020, 32 (4), 1906657 10.1002/adma.201906657. 31814185

[ref6] Wang X.; Mao G.; Ge J.; Drack M.; Cañón Bermúdez G. S.; Wirthl D.; Illing R.; Kosub T.; Bischoff L.; Wang C.; Fassbender J.; Kaltenbrunner M.; Makarov D. Untethered and Ultrafast Soft-Bodied Robots. Commun. Mater. 2020, 1 (1), 67 10.1038/s43246-020-00067-1.

[ref7] Wu S.; Hu W.; Ze Q.; Sitti M.; Zhao R. Multifunctional Magnetic Soft Composites: A Review. Multifunct. Mater. 2020, 3 (4), 042003 10.1088/2399-7532/abcb0c. 33834121 PMC7610551

[ref8] Alapan Y.; Karacakol A. C.; Guzelhan S. N.; Isik I.; Sitti M. Reprogrammable Shape Morphing of Magnetic Soft Machines. Sci. Adv. 2020, 6 (38), eabc6414 10.1126/sciadv.abc6414. 32948594 PMC7500935

[ref9] Bira N.; Dhagat P.; Davidson J. R. A Review of Magnetic Elastomers and Their Role in Soft Robotics. Front. Robot. AI 2020, 7, 588391 10.3389/frobt.2020.588391. 33501346 PMC7805737

[ref10] Jolly M. R.; Carlson J. D.; Muñoz B. C.; Bullions T. A. The Magnetoviscoelastic Response of Elastomer Composites Consisting of Ferrous Particles Embedded in a Polymer Matrix. J. Intell. Mater. Syst. Struct. 1996, 7 (6), 613–622. 10.1177/1045389X9600700601.

[ref11] Rigbi Z.; Jilkén L. The Response of an Elastomer Filled with Soft Ferrite to Mechanical and Magnetic Influences. J. Magn. Magn. Mater. 1983, 37 (3), 267–276. 10.1016/0304-8853(83)90055-0.

[ref12] Bastola A. K.; Hossain M. A Review on Magneto-Mechanical Characterizations of Magnetorheological Elastomers. Composites, Part B 2020, 200, 108348 10.1016/j.compositesb.2020.108348.

[ref13] Borin D.; Stepanov G. Magneto-Mechanical Properties of Elastic Hybrid Composites. Phys. Sci. Rev. 2020, 7 (10), 1119 10.1515/psr-2019-0126.

[ref14] Zhao R.; Kim Y.; Chester S. A.; Sharma P.; Zhao X. Mechanics of Hard-Magnetic Soft Materials. J. Mech. Phys. Solids 2019, 124, 244–263. 10.1016/j.jmps.2018.10.008.

[ref15] Hu W.; Lum G. Z.; Mastrangeli M.; Sitti M. Small-Scale Soft-Bodied Robot with Multimodal Locomotion. Nature 2018, 554 (7690), 81–85. 10.1038/nature25443. 29364873

[ref16] Lum G. Z.; Ye Z.; Dong X.; Marvi H.; Erin O.; Hu W.; Sitti M. Shape-Programmable Magnetic Soft Matter. Proc. Natl. Acad. Sci. U. S. A. 2016, 113 (41), E6007–E6015. 10.1073/pnas.1608193113. 27671658 PMC5068264

[ref17] Manamanchaiyaporn L.; Xu T.; Wu X. Magnetic Soft Robot With the Triangular Head–Tail Morphology Inspired By Lateral Undulation. IEEE/ASME Trans. Mechatron. 2020, 25 (6), 2688–2699. 10.1109/TMECH.2020.2988718.

[ref18] Xu T.; Zhang J.; Salehizadeh M.; Onaizah O.; Diller E. Millimeter-Scale Flexible Robots with Programmable Three-Dimensional Magnetization and Motions. Sci. Robot. 2019, 4 (29), eaav4494 10.1126/scirobotics.aav4494. 33137716

[ref19] Zhang J.; Ren Z.; Hu W.; Soon R. H.; Yasa I. C.; Liu Z.; Sitti M. Voxelated Three-Dimensional Miniature Magnetic Soft Machines via Multimaterial Heterogeneous Assembly. Sci. Robot. 2021, 6 (53), eabf0112 10.1126/scirobotics.abf0112. 34043568 PMC7612277

[ref20] Kotikian A.; McMahan C.; Davidson E. C.; Muhammad J. M.; Weeks R. D.; Daraio C.; Lewis J. A. Untethered Soft Robotic Matter with Passive Control of Shape Morphing and Propulsion. Sci. Robot. 2019, 4 (33), eaax7044 10.1126/scirobotics.aax7044. 33137783

[ref21] White T. J.; Broer D. J. Programmable and Adaptive Mechanics with Liquid Crystal Polymer Networks and Elastomers. Nat. Mater. 2015, 14, 12 10.1038/nmat4433. 26490216

[ref22] Terentjev E. M. Liquid-Crystalline Elastomers. J. Phys.: Condens. Matter 1999, 11, 20 10.1088/0953-8984/11/24/201.

[ref23] López-Valdeolivas M.; Liu D.; Broer D. J.; Sánchez-Somolinos C. 4D Printed Actuators with Soft-Robotic Functions. Macromol. Rapid Commun. 2018, 39 (5), 1700710 10.1002/marc.201700710. 29210486

[ref24] Broer D. J. On the History of Reactive Mesogens: Interview with Dirk J. Broer. Adv. Mater. 2020, 32 (20), 1905144 10.1002/adma.201905144. 31867734

[ref25] de Haan L. T.; Sánchez-Somolinos C.; Bastiaansen C. M. W.; Schenning A. P. H. J.; Broer D. J. Engineering of Complex Order and the Macroscopic Deformation of Liquid Crystal Polymer Networks. Angew. Chem., Int. Ed. 2012, 51 (50), 12469–12472. 10.1002/anie.201205964. 23124726

[ref26] De Haan L. T.; Gimenez-Pinto V.; Konya A.; Nguyen T.-S.; Verjans J. M. N.; Sánchez-Somolinos C.; Selinger J. V.; Selinger R. L. B.; Broer D. J.; Schenning A. P. H. J. Accordion-like Actuators of Multiple 3D Patterned Liquid Crystal Polymer Films. Adv. Funct. Mater. 2014, 24 (9), 1251–1258. 10.1002/adfm.201302568.

[ref27] Broer D. J.; Mol G. N. Anisotropic Thermal Expansion of Densely Cross-Linked Oriented Polymer Networks. Polym. Eng. Sci. 1991, 31 (9), 625–631. 10.1002/pen.760310902.

[ref28] Crawford G. P.; Broer D. J.; Žumer S. In Cross-Linked Liquid Crystalline Systems: From Rigid Polymer Networks to Elastomers, The Liquid Crystals Book Series; CRC Press: Boca Raton, FL, 2011.

[ref29] Warner M. Topographic Mechanics and Applications of Liquid Crystalline Solids. Annu. Rev. Condens. Matter Phys. 2020, 11 (1), 125–145. 10.1146/annurev-conmatphys-031119-050738.

[ref30] Yu Y.; Nakano M.; Ikeda T. Directed Bending of a Polymer Film by Light. Nature 2003, 425 (6954), 145 10.1038/425145a. 12968169

[ref31] Naciri J.; Srinivasan A.; Jeon H.; Nikolov N.; Keller P.; Ratna B. R. Nematic Elastomer Fiber Actuator. Macromolecules 2003, 36 (22), 8499–8505. 10.1021/ma034921g.

[ref32] Herbert K. M.; Fowler H. E.; McCracken J. M.; Schlafmann K. R.; Koch J. A.; White T. J. Synthesis and Alignment of Liquid Crystalline Elastomers. Nat. Rev. Mater. 2022, 7 (1), 23–38. 10.1038/s41578-021-00359-z.

[ref33] Javadzadeh M.; Del Barrio J.; Sánchez-Somolinos C. Melt Electrowriting of Liquid Crystal Elastomer Scaffolds with Programmed Mechanical Response. Adv. Mater. 2023, 35 (14), 2209244 10.1002/adma.202209244. 36459991

[ref34] Lugger S. J. D.; Ceamanos L.; Mulder D. J.; Sánchez-Somolinos C.; Schenning A. P. H. J. 4D Printing of Supramolecular Liquid Crystal Elastomer Actuators Fueled by Light. Adv. Mater. Technol. 2023, 8 (5), 2201472 10.1002/admt.202201472.

[ref35] Ware T. H.; McConney M. E.; Wie J. J.; Tondiglia V. P.; White T. J. Voxelated Liquid Crystal Elastomers. Science 2015, 347 (6225), 982–984. 10.1126/science.1261019. 25722408

[ref36] Guo Y.; Zhang J.; Hu W.; Khan M. T. A.; Sitti M. Shape-Programmable Liquid Crystal Elastomer Structures with Arbitrary Three-Dimensional Director Fields and Geometries. Nat. Commun. 2021, 12 (1), 5936 10.1038/s41467-021-26136-8. 34642352 PMC8511085

[ref37] Ambulo C. P.; Burroughs J. J.; Boothby J. M.; Kim H.; Shankar M. R.; Ware T. H. Four-Dimensional Printing of Liquid Crystal Elastomers. ACS Appl. Mater. Interfaces 2017, 9 (42), 37332–37339. 10.1021/acsami.7b11851. 28967260

[ref38] Kotikian A.; Truby R. L.; Boley J. W.; White T. J.; Lewis J. A. 3D Printing of Liquid Crystal Elastomeric Actuators with Spatially Programed Nematic Order. Adv. Mater. 2018, 30 (10), 1706164 10.1002/adma.201706164. 29334165

[ref39] Sánchez-Somolinos C. 4D Printing: An Enabling Technology for Soft Robotics. In Mechanically Responsive Materials for Soft Robotics; Wiley, 2020; pp 347–360.

[ref40] Ceamanos L.; Kahveci Z.; López-Valdeolivas M.; Liu D.; Broer D. J.; Sánchez-Somolinos C. Four-Dimensional Printed Liquid Crystalline Elastomer Actuators with Fast Photoinduced Mechanical Response toward Light-Driven Robotic Functions. ACS Appl. Mater. Interfaces 2020, 12 (39), 44195–44204. 10.1021/acsami.0c13341. 32885661

[ref41] de Haan L. T.; Verjans J. M. N.; Broer D. J.; Bastiaansen C. W. M.; Schenning A. P. H. J. Humidity-Responsive Liquid Crystalline Polymer Actuators with an Asymmetry in the Molecular Trigger That Bend, Fold, and Curl. J. Am. Chem. Soc. 2014, 136 (30), 10585–10588. 10.1021/ja505475x. 25022765

[ref42] Ceamanos L.; Mulder D. J.; Kahveci Z.; López-Valdeolivas M.; Schenning A. P. H. J.; Sánchez-Somolinos C. Photomechanical Response under Physiological Conditions of Azobenzene-Containing 4D-Printed Liquid Crystal Elastomer Actuators. J. Mater. Chem. B 2023, 11 (18), 4083–4094. 10.1039/D2TB02757G. 37092961

[ref43] Bastola A. K.; Paudel M.; Li L.; Li W. Recent Progress of Magnetorheological Elastomers: A Review. Smart Mater. Struct. 2020, 29 (12), 123002 10.1088/1361-665X/abbc77.

[ref44] Chung H.; Parsons A. M.; Zheng L. Magnetically Controlled Soft Robotics Utilizing Elastomers and Gels in Actuation: A Review. Adv. Intell. Syst. 2021, 3 (3), 2000186 10.1002/aisy.202000186.

[ref45] El-Atab N.; Mishra R. B.; Al-Modaf F.; Joharji L.; Alsharif A. A.; Alamoudi H.; Diaz M.; Qaiser N.; Hussain M. M. Soft Actuators for Soft Robotic Applications: A Review. Adv. Intell. Syst. 2020, 2 (10), 2000128 10.1002/aisy.202070102.

[ref46] Kim Y.; Zhao X. Magnetic Soft Materials and Robots. Chem. Rev. 2022, 122 (5), 5317–5364. 10.1021/acs.chemrev.1c00481. 35104403 PMC9211764

[ref47] Ubaidillah; Sutrisno J.; Purwanto A.; Mazlan S. A. Recent Progress on Magnetorheological Solids: Materials, Fabrication, Testing, and Applications: Recent Progress on Magnetorheological Solids. Adv. Eng. Mater. 2015, 17 (5), 563–597. 10.1002/adem.201400258.

[ref48] Kaiser A.; Winkler M.; Krause S.; Finkelmann H.; Schmidt A. M. Magnetoactive Liquid Crystal Elastomer Nanocomposites. J. Mater. Chem. 2009, 19 (4), 538–543. 10.1039/B813120C.

[ref49] Winkler M.; Kaiser A.; Krause S.; Finkelmann H.; Schmidt A. M. Liquid Crystal Elastomers with Magnetic Actuation. Macromol. Symp. 2010, 291–292 (1), 186–192. 10.1002/masy.201050522.

[ref50] Zhang J.; Guo Y.; Hu W.; Soon R. H.; Davidson Z. S.; Sitti M. Liquid Crystal Elastomer-Based Magnetic Composite Films for Reconfigurable Shape-Morphing Soft Miniature Machines. Adv. Mater. 2021, 33 (8), 2006191 10.1002/adma.202006191. PMC7610459 33448077

[ref51] Zhang J.; Guo Y.; Hu W.; Sitti M. Wirelessly Actuated Thermo- and Magneto-Responsive Soft Bimorph Materials with Programmable Shape-Morphing. Adv. Mater. 2021, 33 (30), 2100336 10.1002/adma.202100336. PMC7612658 34048125

[ref52] Kurniawan C.; Hutahaean R. M.; Muljadi The Effect of Low Vacuum Curing to Physical and Magnetic Properties of Bonded Magnet Pr-Fe-B. Adv. Mater. Res. 2015, 1123, 84–87. 10.4028/www.scientific.net/AMR.1123.84.

[ref53] Magnequench GmbH. Mqp-16-7-20068-070 Data Sheet, 2021, https://mqitechnology.com/wp-content/uploads/2017/09/mqp-16-7-20068-070.pdf (accessed Aug 11, 2023)

[ref54] Vernon-Parry K. D. Scanning Electron Microscopy: An Introduction. III-Vs Rev. 2000, 13 (4), 40–44. 10.1016/S0961-1290(00)80006-X.

[ref55] Modes C. D.; Bhattacharya K.; Warner M. Gaussian Curvature from Flat Elastica Sheets. Proc. R. Soc. Math. Phys. Eng. Sci. 2011, 467 (2128), 1121–1140. 10.1098/rspa.2010.0352.

