# Supplementary material for: Multimodal and Multistimuli 4D-Printed Magnetic Composite Liquid Crystal Elastomer Actuators
Source: ACS Appl Mater Interfaces. 2023 Dec 27;16(2):2704–15. doi: 10.1021/acsami.3c14607 (PMC10797586; doi:10.1021/acsami.3c14607)
Supplement: Supplementary file 1 — am3c14607_si_001.pdf [file am3c14607_si_001.pdf]

Supporting Information:

Multi-Modal and Multi-Stimuli 4D Printed Magnetic Composite Liquid Crystal Elastomer Actuators

*Erick R. Espíndola-Pérez[1], Javier Campo[1], Carlos Sánchez-Somolinos[1,2]**

[1] Instituto de Nanociencia y Materiales de Aragón (INMA), CSIC-Universidad de Zaragoza, Departamento de Física de la Materia Condensada, Zaragoza, 50009, Spain

[2] Centro de Investigación Biomédica en Red de Bioingeniería, Biomateriales y Nanomedicina, Instituto de Salud Carlos III, Zaragoza, 50018, Spain

E-mail: carlos.s@csic.es
* Corresponding author

Keywords: Liquid Crystalline Elastomers, 4D Printing, Magnetic Soft Robots, Multi-Modal Devices, Multi-Stimuli Actuators

Supporting Table

Table S1. Liquid crystal to isotropic transition temperature (Ti) of the macromers including MMPs at different solid contents. Measurements performed by differential scanning calorimetry (DSC).

Solid Content (wt%)	T_i (°C)
0	103.8
10	101.7
25	100.8
50	98.6

Supporting Figures

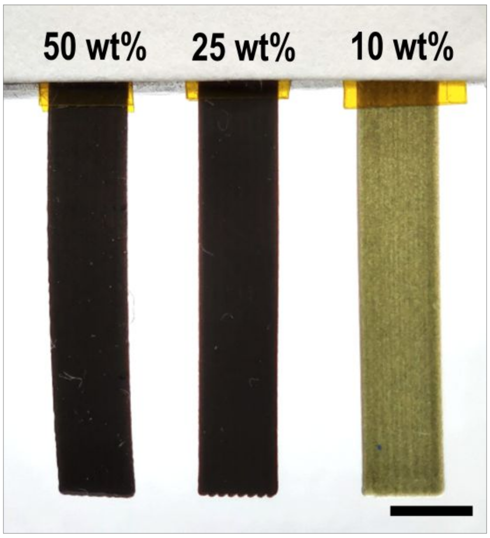

Figure S1. Photograph of free-standing uniaxial actuators printed at different solid contents (scale bar: 5 mm).

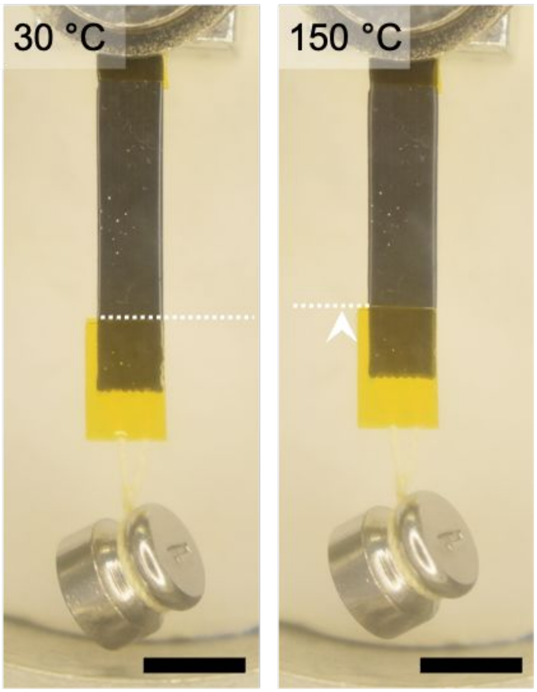

Figure S2. Thermomechanical test of a 50 wt% MMPs uniaxial actuator. The white arrow indicates the contraction along its length.

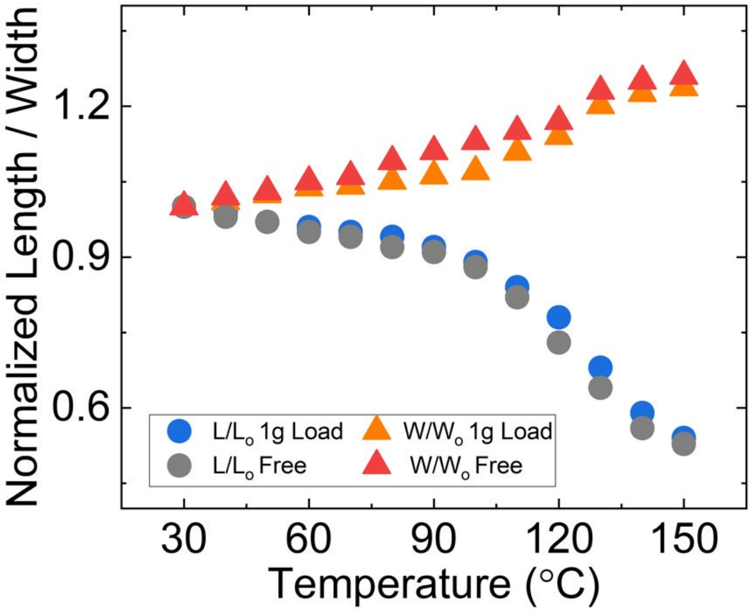

Figure S3. Thermomechanical response of uniaxial actuator (85 μm) at 0 wt% MMPs with 1 g load and load free.

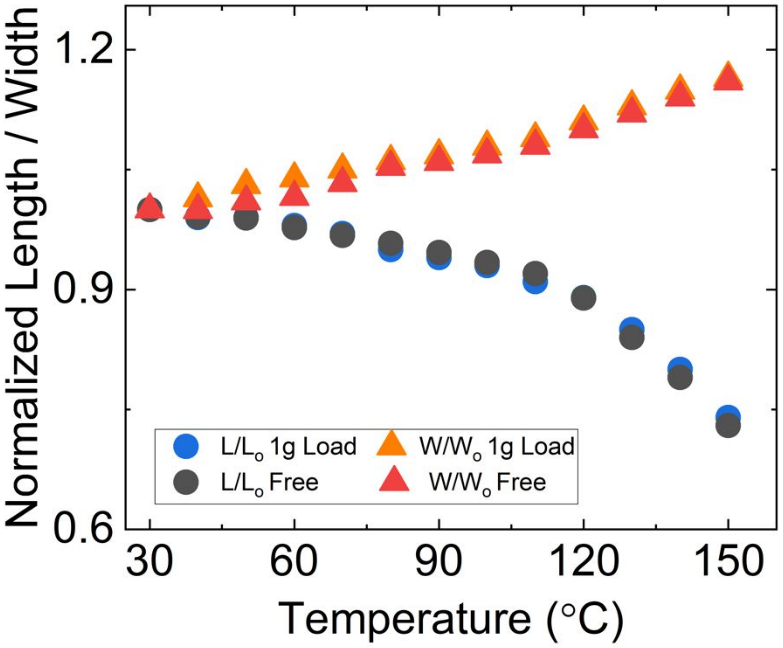

Figure S4. Thermomechanical response of uniaxial actuator (110 μm) at 10 wt% MMPs with 1 g load and load free.

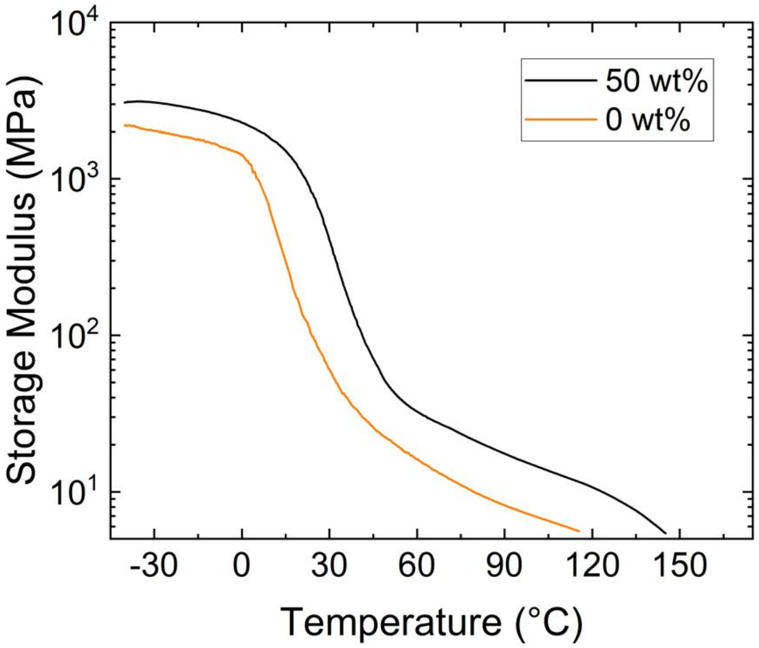

Figure S5. DMA storage modulus (E') of LCE uniaxial actuator at 0 wt% MMPs (105 μm) and 50 wt% MMP uniaxial MLCE actuator (110 μm)

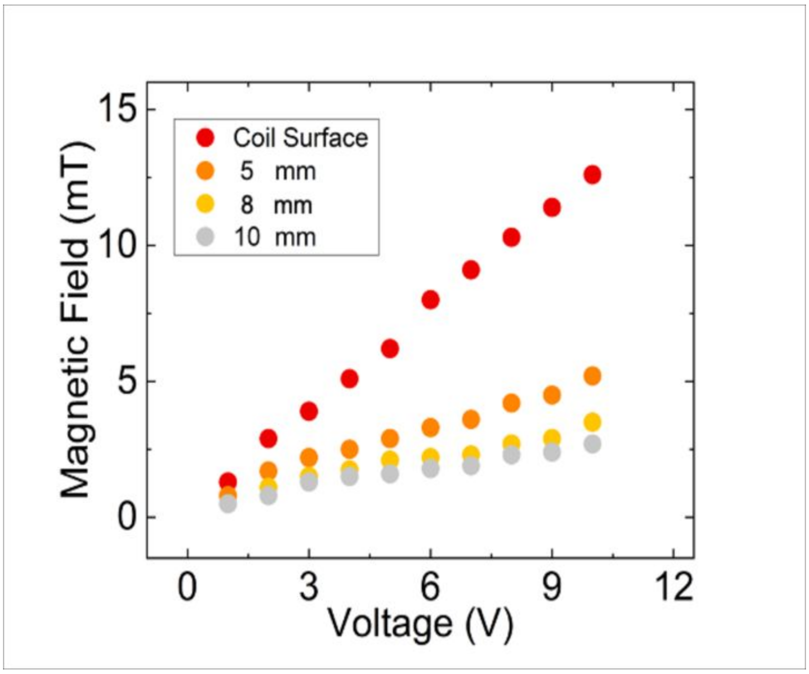

Figure S6. Magnetic field generated by the coils used in Figure 4 and Figure 5, measured at different distances from the surface of the coil.

Supplementary Note 1. Experimental conditions for thermal and multi-stimuli tests

The thermal response studies of the MLCE elements, described throughout this work, was carried out placing the samples in an oven cavity, with a glass cover to provide optical access and retain heat. The thermal process initiates from RT until 110 °C, with a slope of 2.5 °C/min, pictures were taken at 5 °C intervals and later used for the characterization of the system. The supplementary movies S1 and S2 show the real time thermo-actuation of MLCE elements, they are performed in the same oven cavity as described above. The oven was set at 110 °C (measured with the glass cover closed), then samples are placed inside and the glass cover is shut rapidly. The elements are quickly heated to the cavity temperature, responding with an increasing curvature. In the same manner, when the glass cover is removed (Movie S1) the temperature in the cavity volume quickly drops close to 50 °C, cooling down the sample and returning to a lower curvature deformation.

In movies S3 and S4, the elements are placed in a custom-made heating cavity, that allows for close proximity of the magnetic coils, allowing for multi-stimuli actuation, via heat and magnetic field being applied simultaneously. In Movie S3, the bilayer element is heated at 60 °C and AC magnetic field is applied using a pair of coils coaxially aligned, inducing an oscillating motion that can be addressed around the point where the thermal deformation was set. In Movie S4, the same cavity and coils are used to control a beam-steering device, enabled by the multi-stimuli and multi-modal capabilities of MLCEs. The pair of coils are now orthogonal to each other and magnetic field is produced in or out of phase and with varying frequencies independently. This allows to control the beam via torsion and bending motion induced by magnetic fields, allowing for X and Y beam directionality, respectively.

Supporting Movies

Movie S1. Thermal Actuation, of a Magnetically Responsive Liquid Crystal Elastomer, 4D Printed Element Bilayer. A 4D printed bilayer element of 50 wt% MMPs is placed in an oven cavity heated at 110 °C to show the thermally-induced deformation, programmed via 4D printing to increase its curvature, forming a loop. When cooled down, the element returns to low curvature deformation.

Movie S2. Thermal Actuation, of a Magnetically Responsive Liquid Crystal Elastomer, 4D Printed Spiral-Like Element. The thermally-induced deformation of a printed pattern, similar to an azimuthal director field around a +1 disclination, programmed via 4D printing. The MLCE element of 50 wt% MMPs, is placed in on top of an aluminum stage inside an oven cavity heated at 110 °C. The element increases its curvature, forming a conical shape.

Movie S3. Magnetic and Thermal Actuation of a Bilayer Actuator of Magnetically Responsive Liquid Crystal Elastomers. Multi-stimuli and multimodal behavior, induced by magnetic and thermal stimuli, of a MLCE bilayer actuated at 60 °C with a sine wave magnetic input at ±10 mT peak and a frequency of 5 Hz. The initial position of the oscillating motion is controlled by the temperature-induced curvature.

Movie S4. Multi-Stimuli, Multi-Mode Beam Steering Device Directing a Beam on the XY axis and Examples of Lissajous Patterns. Demonstration of the capabilities of the beam steering device. The beam is directed along the X and Y axes, using one input only. After, the use of simultaneous inputs creates beam patterns, as shown for a straight line tilted 45° with a phase shift of 0° and a 1:1 X:Y frequency ratio [0°, 1:1]. Followed by a curved pattern using out of phase (45°) magnetic fields with a 1:2 frequency ratio [45°,1:2].
